# Total Electrosynthesis of N, N‐Dimethylformamide From CO_2_ and NO_3_^−^

**DOI:** 10.1002/advs.202414431

**Published:** 2024-11-21

**Authors:** Shuai Yan, Shuai Chen, Morgan McKee, Alexandre Terry, Ralf Weisbarth, Nikolay Kornienko

**Affiliations:** ^1^ Institute of Inorganic Chemistry University of Bonn Gerhard‐Domagk‐Str. 1 53121 Bonn Germany

**Keywords:** coupling, electrosynthesis, hydrogenation, retrosynthetic analysis

## Abstract

Electrochemical C−N coupling presents a promising strategy for converting abundant small molecules like CO_2_ and NO_3_^−^ to produce low‐carbon‐intensity chemicals in a potentially more sustainable route. A prominent challenge is the limited product scope, particularly for organonitrogen chemicals featuring a variety of functional groups, alongside the limited understanding of plausible reaction mechanisms leading up to these products. In light of this, the total electrosynthesis method is reported for producing N, N‐dimethylformamide (DMF), a widespread solvent and commodity chemical, from NO_3_^−^ and CO_2_. This method enabled a notable production rate of 1.24 mmol h^−1^ g_cat_^−1^ for DMF employing a hybrid Ag/Cu catalyst. Additionally, an impressive Faradaic efficiency (FE) of 28.6% is attained for DMF through oxidative coupling of dimethylamine using Ag/Cu catalyst. Through a distinctive retrosynthetic experimental analysis, the DMF synthesis pathway is systematically deconstructed, tracing its origins from dimethylamine to methylamine, and ultimately to CO_2_ and NO_3_^−^. The investigation revealed that the hydrogenation of coupled intermediates proves to be the limiting step, rather than the C−N coupling steps in the synthetic pathway. Finally, using a combination of in situ measurements and retrosynthetic analysis, the possible mechanism is elucidated underlying DMF synthesis and identified subsequent routes for system improvement.

## Graphical abstract

A sustainable electrosynthesis method using renewable energy converts CO_2_ and NO_3_^−^ directly into N, N‐dimethylformamide, a widespread solvent and commodity chemical, thereby providing a promising approach to reduce greenhouse gas emissions and NO_3_^−^ waste. This work also systematically deconstructs the DMF synthesis pathway, tracing its origins from dimethylamine to methylamine, and ultimately back to CO_2_ and NO_3_^−^ as feedstocks.

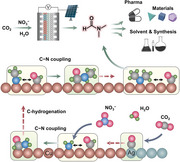

## 1 Introduction

C−N bond‐containing products are essential across various sectors such as agriculture, pharmaceuticals, organic synthesis, and plastics.^[^ ^1^ ^]^ Urea, for instance, acts as a vital nitrogen‐releasing fertilizer,^[^ ^2^ ^]^ while other amino compounds play critical roles in pharmaceuticals and medical materials.^[^ ^3^ ^]^ Acrylonitrile is indispensable in the production of plastics and synthetic fibers, constituting a huge market.^[^ ^4^ ^]^ However, industrial routes of these chemicals predominantly depend on NH_3_ as a precursor, which is produced via the Haber‐Bosch process,^[^ ^5^ ^]^ followed by thermochemical amination or ammoxidation,^[^ ^6^, ^7^ ^]^ thus contributing substantially to greenhouse gas (GHG) emissions. Electrosynthetic routes using Earth‐abundant CO_2_ and NO_3_^−^ as building blocks offers a potentially greener approach for producing low‐carbon‐intensity chemicals.^[^ ^8^, ^9^ ^]^ Recent advancements have demonstrated the successful synthesis of various C−N compounds such urea,^[^ ^10^, ^11^ ^]^ amines (methylamine, ethylamine)^[^ ^12^, ^13^ ^]^ and amides (formamide, acetamide)^[^ ^14^, ^15^ ^]^ through CO_2_‐integrated electrocatalytic NO_2_^−^/NO_3_^−^ reduction, making the synthesis of more complex C−N compounds highly promising. Current research efforts are primarily focused on designing dual‐site and heterojunction catalysts that can effectively generate and balance the surface intermediates containing carbon and nitrogen, while simultaneously facilitating C−N coupling.^[^ ^10^, ^16^ ^]^ A variety of coupling mechanisms have been proposed including *CO/*COOH and *NH_2_,^[^ ^11^, ^16^ ^]^ *CO and *NO,^[^ ^17^ ^]^ *NH and CO_2_/*COOH/*CO,^[^ ^18^ ^]^ NH_2_OH and HCHO,^[^ ^9^ ^]^ etc. However, the exact mechanisms underlying these processes remain an open question in the community,^[^ ^16^ ^]^ and this uncertainty contributes to the challenge of increasing product scope.

**Figure 1:**
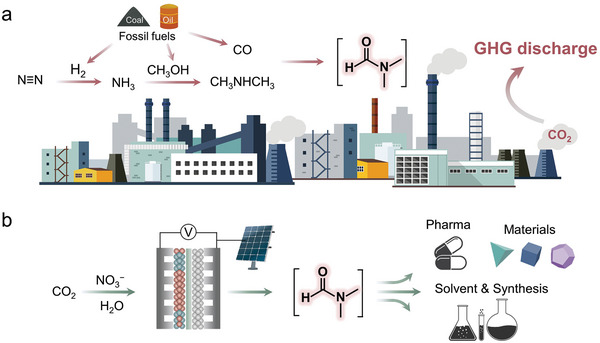
Conventional thermochemical methods for producing DMF involve multiple reaction steps, each generating significant GHG emissions and consuming fossil energy sources a). Using renewable energy for the electrochemical direct conversion of CO_2_ and NO_3_^−^ to produce DMF and its importance in pharmaceuticals, organic synthesis, materials, and solvent applications b).

N, N‐Dimethylformamide (DMF), widely used as a polar aprotic and hydrophilic solvent, serves as an important repository of building blocks for various functional groups (e.g., H, CHO, HCO_2_, CO, N(CH_3_)_2_) in organic synthesis, and also plays a crucial role in pharmaceutical and materials synthesis (**Figure** **1**).^[^ ^19^, ^20^, ^21^ ^]^ The global market for DMF reached $2.3 billion in 2022, with an anticipated growth to ≈$2.7 billion by 2027.^[^ ^20^ ^]^ Currently, DMF synthesis predominantly relies on thermocatalytic conversion of CO and dimethylamine under harsh conditions, requiring high pressures (0.5–11.0 MPa) and temperatures (323−473 K) (Figure 1a).^[^ ^20^, ^22^ ^]^ In this process, CO and H_2_ are primarily produced via natural gas reforming or coal gasification,^[^ ^23^, ^24^ ^]^ while dimethylamine is derived from the methylation of NH_3_ with methanol.^[^ ^25^ ^]^ NH_3_ is produced via the Haber‐Bosch process and methanol is synthesized from syngas using fossil fuels as feedstock (Figure 1a).^[^ ^5^, ^26^ ^]^ Consequently, the synthesis of DMF entails many steps, each of which undoubtedly contributes significantly to its carbon footprint.

DMF can also be synthesized via electrochemical strategies.^[^ ^20^, ^27^, ^28^ ^]^ Recently, Wang and colleagues used dimethylamine and CO_2_ precursors to synthesize DMF with a FE (FE_DMF_) of 37.5%.^[^ ^20^ ^]^ Similarly, Duan et al. achieved a FE_DMF_ exceeding 50% by oxidizing trimethylamine.^[^ ^27^ ^]^ Zhong and co‐workers also developed a WO_2_−NiOOH/Ni composite catalyst that achieved an impressive FE_DMF_ of ≈50% using dimethylamine and methanol as reactants.^[^ ^28^ ^]^ Nevertheless, these routes still depended on amine chemicals as reactants. Developing electrosynthetic routes of DMF from CO_2_ and NO_3_^−^ presents a potentially more sustainable strategy to reduce GHG emissions and NO_3_^−^ waste. A prominent challenge lies in understanding the fundamental principles of this process to enable DMF formation while simultaneously minimizing side reactions including CO_2_, NO_3_^−^ and H_2_O reduction.^[^ ^29^ ^]^ A substantial challenge is that the synthesis of DMF entails a complex series of reactions involving the transfer of 22 electrons and 23 protons per product molecule. This includes multiple hydrogenation and coupling steps:

(1) $$3CO_{2}+22e^{-}+23H^{+}+{\mathrm{NO}}_{3}^{-}\to \mathrm{HCON}{\left(CH_{3}\right)}_{2}+8H_{2}O$$

This complexity may elucidate why DMF has not yet been successfully synthesized from CO_2_ and NO_3_^−^.

In this study, we introduce a total electrosynthesis of DMF from NO_3_^−^ and CO_2_, enabling a high production rate of 1.24 mmol h^−1^ g_cat_^−1^ for DMF production using a hybrid Ag/Cu catalyst. We also achieved a FE_DMF_ up to 28.6% from the oxidative C−N coupling using Ag/Cu catalyst. Systematic experiments corroborate the origin of DMF from CO_2_ and NO_3_^−^, while revealing remarkably high coupling efficiency between hydrogenated C−N precursors and CO_2_. This prompts an investigation into C−N coupling or hydrogenation serves as the limiting step in DMF synthesis. We systematically designed retrosynthetic routes using hydrogenated carbon and nitrogen sources to dissect the DMF synthesis. Our experimental results demonstrated that hydrogenation, rather than C−N coupling, dictated the reaction rate in our system. We also used in situ electrochemical mass spectrometry (ECMS) and in situ attenuated total reflection surface‐enhanced infrared absorption spectroscopy (ATR‐SEIRAS) to comprehensively elucidate reaction mechanisms. Crucially, we not only expand the product scope of sustainable C−N electrosynthesis but also give new sight by emphasizing the enhancement of hydrogenation efficiency rather than the conventional focus on improving coupling efficiency.

## 2 Material Synthesis and Electrocatalysis

We first prepared the hybrid Ag/Cu catalyst which is known to prefer CO_2_ reduction reaction (CO_2_RR) and NO_3_^−^ reduction reaction (NO_3_RR) over hydrogen evolution reaction (HER).^[^ ^29^ ^−^ ^31^ ^]^ Based on X‐ray diffraction (XRD) analysis, the metallic phases of Ag/Cu catalyst exhibited predominant crystalline facets of Cu(111), Ag(111), and Ag(002) (Figure S1, Supporting Information). Scanning electron microscopy (SEM) and transmission electron microscopy (TEM) investigations revealed Cu and Ag nanoparticles formed distinct Cu and Ag layers (Figures S2 and S3, Supporting Information). The approximate thicknesses of the Ag and Cu layers were determined using inductively coupled plasma optical emission spectroscopy (ICP‐OES) (Figure S4 and Table S1, Supporting Information).

**Figure 2:**
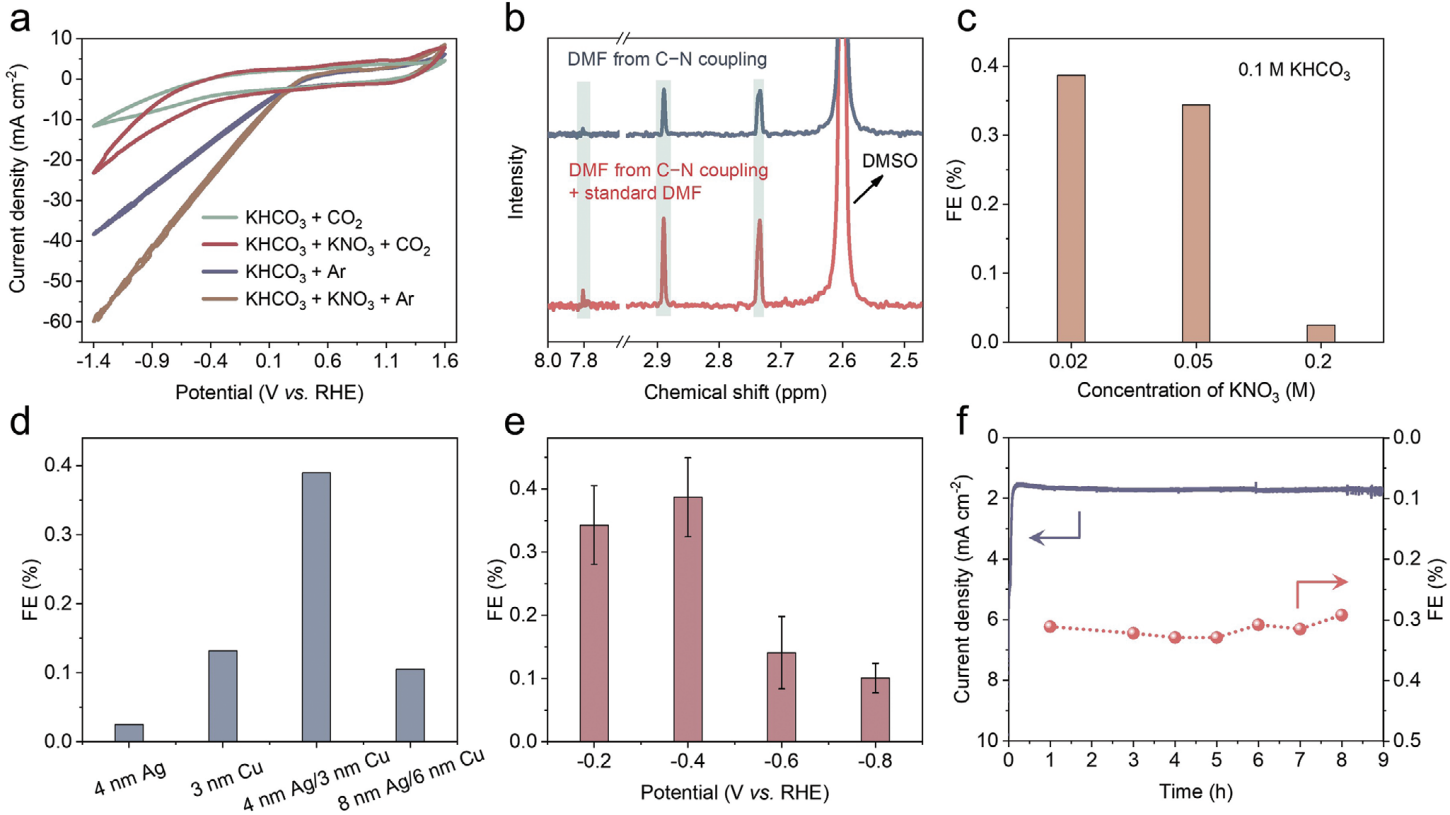
Electrochemical C−N coupling performances. The presence of CO_2_ effectively suppresses HER and NO_3_RR, enabling selective C−N bond formation a). The identification of DMF synthesized from CO_2_ and NO_3_^−^ is demonstrated b). Adjusting the concentration of NO_3_^−^ enhances selectivity toward  DMF c). Reducing the thickness of both Ag and Cu metals increases the exposure of Ag sites, thereby promoting DMF generation d). Potential‐dependent FE values for DMF production using an Ag/Cu hybrid catalyst are presented e). Consistent DMF production over time is maintained at −0.4 V f).

We then evaluated the DMF electrosynthesis performance using a gas‐diffusion electrode (GDE) half‐cell (Figure S5, Supporting Information), which offers two advantages: minimal electrolyte volume allows detection even at low yields, and a pressure gradient between the CO_2_ flow side and the electrolyte side prevents flooding issues, enabling prolonged operation. In this study, all applied potentials were adjusted to the reversible hydrogen electrode (RHE) scale, without applying iR‐correction. As seen in cyclic voltammetry (CV) curves, the addition of KNO_3_ to KHCO_3_ resulted in a significant increase in current density, attributed to NO_3_RR (**Figure** **2**a). The introduction of CO_2_ into the KNO_3_ system reduced the total current density by suppressing both NO_3_RR and HER, thus favoring the formation of C−N bonds. Nonetheless, the current density in the CO_2_ and KNO_3_ system remained higher than in the CO_2_‐only system, due to the C−N coupling and NO_3_RR. Next, we used ^1^H‐NMR and gas chromatography to quantify liquid and gas products (Figures S6−S9, Supporting Information). By adding a commercial DMF aqueous solution to the product mixture, we observed an increase in the C−N coupling peaks, providing an initial confirmation of DMF formation (Figure 2b). In order to balance NO_3_RR and CO_2_RR to push DMF selectivity, we optimized the concentration of electrolytes and selected 0.02 m KNO_3_ as higher NO_3_^−^ concentrations could favor NH_3_ production (Figure 2c).

We next investigated the effects of the catalyst's composition and structure on the performance of DMF formation. The 4 nm Ag/3 nm Cu catalyst showed the highest FE_DMF_ at 0.39%, surpassing that of single‐component 3 nm Cu (0.13%) and 4 nm Ag (0.025%) at −0.4 V (Figure 2d). However, with an 8 nm Ag/6 nm Cu catalyst, the FE_DMF_ decreased to 0.11%, similar to that of Cu alone. We tentatively attribute this to the increased the thickness of the Cu and Ag layers reducing the exposure of Ag sites, thereby significantly reducing the activation effect of CO_2_ to adsorbed *CO by Ag. The optimized 4 nm Ag/3 nm Cu catalyst exhibited a potential‐dependent product distribution (Figure 2e), with the FE_DMF_ peak at −0.4 V, corresponding the peak partial current density (j_DMF_) of 4.67 µA cm^−2^ (Figure S10, Supporting Information). The production rate of DMF can reach 1.24 mmol h^−1^ g_cat_^−1^ corresponding to 9.2 nmol h^−1^ cm^−2^ at −0.8 V (Figures S10 and S11, Supporting Information). This hybrid Ag/Cu electrocatalyst also exhibited high stability over 9 h at −0.4 V, with a stable current density of ≈1.80 mA cm^−2^ and a FE_DMF_ of ≈0.32% (Figure 2f). Compared to previous reports,^[^ ^20^, ^27^, ^28^ ^]^ our performance may not be competitive, but this approach aims to synthesize complex C−N products from simple substrates (CO_2_ and NO_3_^−^) and enhances our understanding of the underlying mechanisms.

**Figure 3:**
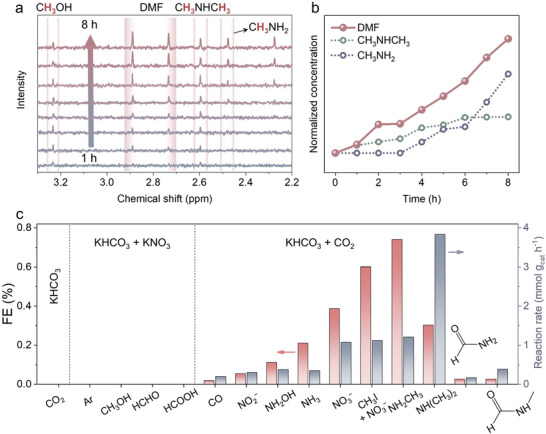
Electrochemical mechanism investigations. Product distribution of C−N coupling from CO_2_ and NO_3_^−^ over time a). Variation in DMF concentration over time correlates with changes in methylamine and dimethylamine concentrations b). Control experiments for exploring C−N coupling mechanism using different carbon sources and nitrogen sources c).

We also conducted a long‐time C−N electrosynthesis, analyzing the concentration of products at different time intervals and their interdependencies (**Figure** **3**a,b). The concentration of DMF increased linearly over time (Figure 3b) without any attenuation. Interestingly, we observed that the concentrations of methylamine also increased linearly, following the same trend as DMF formation. In contrast, the concentration of dimethylamine peaked and then remained constant (Figure 3b; Figure S12, Supporting Information). This suggests that DMF was produced through the sequential upgrading of CO_2_ and NO_3_^−^ to methylamine‐related intermediates, which were then further upgraded to dimethylamine‐related intermediates. To exclude the possibility of DMF formation from the oxidation of amine‐based organic compounds, we conducted C−N coupling at −0.4 V using 0.1 m KHCO_3_ with methylamine, dimethylamine, and trimethylamine, respectively, with their concentrations consistent to those in Figure 3a. Trace DMF peaks were observed only when trimethylamine was used, and the concentrations of these amine intermediates remained nearly unchanged (Figure S13, Supporting Information) due to their low concentration and the poor activity of the graphite anode. Additionally, using a cation‐exchange membrane to separate the cathode and anode did not prevent DMF formation, confirming it originates from the reduction side (Figure S14, Supporting Information).

To further strengthen our findings, we conducted systematic control experiments. Our results demonstrated that DMF formation requires both carbon and nitrogen sources, along with the catalyst, as no DMF was produced in their absence (Table S2, Supporting Information). Additionally, we tested various carbon sources (e.g., methanol, formaldehyde, formic acid, and CO) to determine if they could replace CO_2_ in DMF production (Figure 3c; and Table S2, Supporting Information). We found that DMF products can be generated using CO as substract. However, formic acid and methanol are not effective in providing *CHO and *CH_3_, and formaldehyde tends to be reduced into methanol instead of generating *CHO through dehydrogenation.^[^ ^32^, ^33^ ^]^ We also investigated various nitrogen sources (e.g., NO_2_^−^, NH_2_OH, NH_3_, and NO_3_^−^), all of which produced DMF even if the FE_DMF_ remained low (Figure 3c), with corresponding reaction rates ranging from 0.30 to 1.07 mmol g_cat_^−1^ h^−1^. It is clear that the use of hydrogenated nitrogen sources did not enhance DMF selectivity, which may be attributed to the ease of hydrogenation of NO_3_^−^ to produce various N‐containing intermediates.

Next, we explored the utilization of hydrogenated carbon sources (Figure 3c). Introducing iodomethane into the system increased the reaction rate to 1.12 mmol g_cat_^−1^ h^−1^, and substituting NO_3_^−^ with methylamine further boosted the reaction rate to 1.2 mmol g_cat_^−1^ h^−1^, rising to 3.83 mmol g_cat_^−1^ h^−1^ with dimethylamine with corresponding FE_DMF_ between 0.3% and 0.74%. Compared to methylamine, NH(CH_3_)_2_ may react with *CO or *COOH intermediate to produce DMF, thus requiring fewer methylation steps since it already contains the N(CH_3_)_2_, which may account for the higher DMF reaction rate. However, substituting NO_3_^−^ with N‐methylformamide or formamide resulted in decreases in DMF selectivity to below 0.03% and in the reaction rate to below 0.4 mmol g_cat_^−1^ h^−1^. This could be attributed to the slow hydrogenation process of carbon sources.

## 3 Retrosynthetic Reaction Analysis

In the C−N coupling reaction, our systematic experiments demonstrated that hydrogenation seems to be the determining step. To distinguish between hydrogenation and coupling, we employed retrosynthetic routes, tracing DMF back to dimethylamine, then to methylamine, and ultimately to CO_2_ and NO_3_^−^. We began with dimethylamine as a starting reagent because its conversion to DMF was faster than N‐methylformamide and thus, it was the more likely precursor.

**Figure 4:**
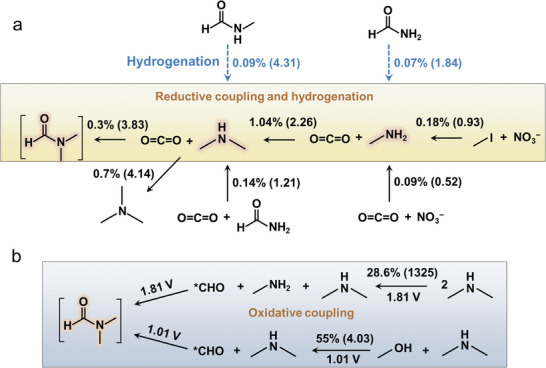
Retrosynthetic reaction analysis. The reductive hydrogenation and coupling reactions of various precursors to produce methylamine, dimethylamine, trimethylamine and DMF products on Ag/Cu catalyst a). The oxidative coupling reactions using Ag/Cu catalyst from dimethylamine, and methanol and dimethylamine, respectively. b). The reaction rates in parentheses are expressed in mmol g_cat_^−1^ h^−1^.

Using dimethylamine and CO_2_ as building blocks, we achieved a reaction rate of 3.83 mmol g_cat_^−1^ h^−1^ and a FE of 0.3% for the production of DMF. In comparison, trimethylamine formation exhibited a higher reaction rate of 4.14 mmol g_cat_^−1^ h^−1^ and a FE of 0.7% (**Figure** **4**a). We then evaluated three potential routes to synthesize dimethylamine. Direct hydrogenation of N‐methylformamide, which involves fewer hydrogenation steps, produced dimethylamine with a FE of 0.09% but a high reaction of 4.31 mmol g_cat_^−1^ h^−1^. Using CO_2_ and formamide as reactants produced a FE of 0.14% and 1.21 mmol g_cat_^−1^ h^−1^ for dimethylamine (Table S3, Supporting Information). Interestingly, replacing formamide with methylamine increased the FE to 1.04% and the rate to 2.26 mmol g_cat_^−1^ h^−1^. This provided evidence that dimethylamine may stem from the coupling between a methylamine fragment and *CH_x_O fragment. Following this, we formulated three approaches to synthesize methylamine. Direct hydrogenation of formamide enabled a FE of 0.07% and a reaction rate of 1.84 mmol g_cat_^−1^ h^−1^. Using CO_2_ and NO_3_^−^ as reactants gave a FE of 0.09% and a rate of 0.52 mmol g_cat_^−1^ h^−1^. Remarkably, using iodomethane, which generates *CH_x_ surface fragments,^[^ ^34^ ^]^ enhanced the FE to 0.18% and the reaction rate to 0.93 mmol g_cat_^−1^ h^−1^. As the reduction of NO_3_^−^ to *NH_x_ fragments proceeds readily on Cu surfaces, the hydrogenation of the carbon fragments seems to be consistently limiting the reaction process, underscoring the critical role of hydrogenation. In the retrosynthesis routes, we applied the same conditions (e.g., potential, catalyst) for the retrosynthetic pathway as for DMF formation to ensure comparability. This approach limited our ability to optimize each reaction, resulting in low selectivity and reaction rates. Additionally, competitive side reactions complicate C−N experiments, making it difficult to promote both hydrogenation and coupling effectively.

In a complementary route to probe C−N coupling en route to DMF synthesis, we further investigated oxidative coupling of C−N bonds from dimethylamine using Ag/Cu and Pt catalysts. DMF and methylamine were produced as the major products, achieving a FE_DMF_ up to 28.6% on Ag/Cu with a reaction rate for DMF of 1325 mmol g_cat_^−1^ h^−1^ at 1.81 V (Figure 4b; and Figure S15 and Table S4, Supporting Information). The Pt catalyst enabled an even higher FE for DMF, reaching 40.3% at 1.61 V (Figures S16 and S17 and Table S5, Supporting Information). Both ^1^H‐NMR and mass spectrometry (MS) analyses confirmed the formation of DMF (Figures S17 and S18, Supporting Information). In this reaction, dimethylamine provides both the carbon and nitrogen sources (Figure S19, Supporting Information), and the overall reaction is as follows:

(2) $$\begin{array}{ccc} 2 & & CH_{3}\mathrm{NHC}H_{3}+H_{2}O\to \mathrm{HCON}{\left(CH_{3}\right)}_{2}\\ & & +CH_{3}NH_{2}+4e^{-}+4H^{+} \end{array}$$

We also achieved oxidative coupling to produce DMF using methanol as the carbon source to provide *CHO species at less oxidizing potentials on Pt and Ag/Cu catalysts. To minimize interference from the self‐oxidation of dimethylamine in DMF production, we performed CV analyses for the oxidations of methanol and dimethylamine on a Pt catalyst, respectively (Figure S20, Supporting Information). We observed that methanol oxidation occurred at a lower overpotential compared to dimethylamine (Figure S20, Supporting Information). Then, we achieved a FE_DMF_ of 18% at 0.86 V using Pt catalyst (Table S5, Supporting Information). Additionally, with the Ag/Cu catalyst, we attained an even higher FE for DMF of 55% at 1.01 V, with a reaction rate of 4.03 mmol g_cat_^−1^ h^−1^ (Figure 4b; and Table S4, Supporting Information). This further demonstrated the fast oxidative coupling when each fragment is fully hydrogenated.

Our retrosynthetic analysis indicates that the hydrogenation process rather than coupling serves as the limiting step in DMF synthesis. Once the initial C−N coupling reaction occurs, further hydrogenation steps are necessary, potentially leading all the way to methylamine or dimethylamine but they are rare products. Instead, urea is more commonly produced. Many studies have reported high urea selectivity (>70%) at low overpotentials.^[^ ^9^, ^10^ ^]^ Our results provide a possible explanation for this: urea can be readily produced without complex hydrogenation processes. This significantly enhances our understanding of electrochemical C−N synthesis, potentially facilitating the expansion of scope to valuable amine and amide products beyond urea.

## 4 Mechanistic Investigations: In Situ Techniques

**Figure 5:**
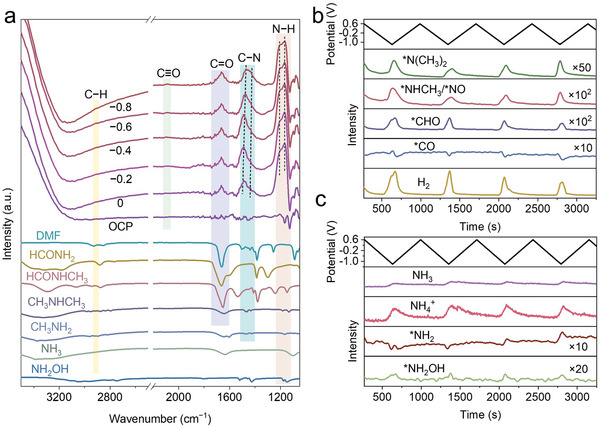
In situ characterization. In situ ATR‐SEIRAS on Ag/Cu catalyst at different potentials in CO_2_‐saturated KHCO_3_ and KNO_3_ electrolytes represented in absorbance units a). FTIR spectra of standard samples are shown at the bottom of the figure for reference, plotted based on their transmission (a). In situ ECMS for the C−N coupling reaction over Ag/Cu catalyst b,c).

We also conducted in situ ATR‐SEIRAS experiments to detect key intermediates (Figure S21, Supporting Information). Introducing only CO_2_ into the system, peaks corresponding to linearly bound *CO species (*CO_atop_, ≈2080 cm^−1^) were observed (Figure S22, Supporting Information).^[^ ^31^ ^]^ When only NO_3_^−^ was present, intermediates such as *NH_2_OH, *NH_2_, and *NO_2_ were detected (Figure S22 and Table S6, Supporting Information).^[^ ^35^, ^36^ ^]^ Upon simultaneous addition of CO_2_ and NO_3_^−^, the intensity of the *CO_atop_ peak decreased significantly, indicating that NO_3_RR reduces the surface coverage of these carbon species (**Figure** **5**a). Additionally, five sets of vibration peaks in this system were observed in this system: C−H vibration (≈2927 cm^−1^),^[^ ^37^ ^]^ C≡O vibration (≈2080 cm^−1^),^[^ ^31^ ^]^ C═O vibration (≈1655 cm^−1^),^[^ ^38^ ^]^ C−N vibration (≈1497 cm^−1^, ≈1436 cm^−1^),^[^ ^39^ ^]^ and N−H vibration (≈1198 cm^−1^, ≈1169 cm^−1^),^[^ ^35^, ^36^ ^]^ respectively (Figure 5a; and Table S6, Supporting Information). To better assign these peaks, we used Fourier‐transform infrared spectroscopy (FTIR) to test a series of standard samples (NH_2_OH, NH_3_, methylamine, dimethylamine, formamide, N‐methylformamide and DMF), which contain C−N, C−H, and C═O bonds similar to those of various intermediates in our system (Figure 5a). We observed C−H peaks at positions similar to those of DMF, N‐methylformamide, and methylamine; C═O peaks at positions similar to those of DMF, N‐methylformamide, and formamide; and N−H peaks at positions similar to those of NH_2_OH, methylamine, dimethylamine, and N‐methylformamide.

Interestingly, we observed that the intensity of peaks at lower frequencies (≈1436 cm^−1^) relative to C−N peaks (≈1497 cm^−1^) increased as the overpotential increases (Figure 5a). This occurred as higher overpotentials accelerated the rates of hydrogenation, resulting in the formation of more reduced C−N coupling intermediates. These intermediates exhibited increased complexity and higher molecular weights, containing more atoms and chemical bonds, thus displaying lower frequencies of C−N derived vibrational modes and correspondingly smaller wavenumbers.^[^ ^40^ ^]^ We also carried out in situ ATR‐SEIRAS experiments using isotope‐labeled ^13^CO_2_ and ^15^NO_3_^−^, respectively, comparing them with CO_2_ and NO_3_^−^ (Figure S23, Supporting Information). Compared to CO_2_ and NO_3_^−^, we can see that C═O peak for CO_2_ and ^15^NO_3_^−^ measurements did not shift, but the C−N and N−H peaks shifted to lower frequencies due to the isotope effect.^[^ ^36^ ^]^ Furthermore, when using ^13^CO_2_ and NO_3_^−^, the C═O and C−N peaks shifted to lower frequencies, while N−H peaks shifted to higher frequencies compared to CO_2_ and ^15^NO_3_^−^. These results further support our peak assignments.

Next, we carried out in situ ECMS measurements to further identify possible intermediates (Figure S24, Supporting Information). We observed the following *m*/*z* signals with a tentative assignment: H_2_ (2); NO (30), NH_2_OH (33), NH_2_ (16), NH_3_ (17), NH_4_^+^ (18); CO (28), CHO (29); NHCH_3_ (30), N(CH_3_)_2_ (44) (Table S7, Supporting Information). Given that some signals are fragments rather than full species, they may represent intermediates or fragments of intermediate products. To validate and support our findings, we also referenced relevant literature and provided attributions in Table S7 (Supporting Information). As seen in Figure 5b,c and Figure S25 (Supporting Information), H_2_ and NH_3_/NH_4_^+^ were the major products. For NO_3_^−^ reduction pathway, it may follow NO_3_^−^ → *NO → *NH_2_OH → *NH_2_ → NH_3_/NH_4_^+^. The *NH_2_ and *NH_2_OH, presented in small intensities, likely participated in the coupling reaction (Figure 5c). However, based on the control experiment in Figure 3c, NH_2_OH showed low performance in producing DMF, suggesting that *NH_2_ may be an intermediate in the reaction. Concurrently, CO_2_ was reduced to *CO and subsequently to *CHO (Figure 5b). Notably, the fragments of methylamine and dimethylamine also support the mechanism from CO_2_ to CH_3_NH*, which is then upgraded to (CH_3_)_2_N*, and finally couples with *CHO to yield the DMF product (Figure 5b).

**Figure 6:**
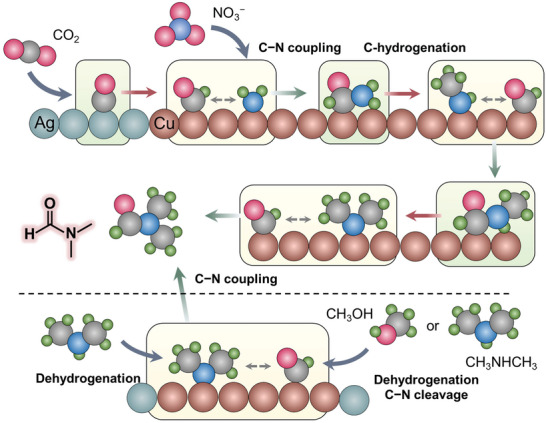
Schematic of DMF formation from reductive (above the dashed line) and oxidative (below the dashed line) coupling on Ag/Cu catalyst, involving multiple slow C‐hydrogenation steps and fast coupling steps.

Finally, based on the above analysis, we propose a possible mechanism for the formation of DMF. In our particular reductive coupling system, *CO is primarily generated on Ag sites from CO_2_ reduction (**Figure** **6**).^[^ ^30^ ^]^ Cu plays a bifunctional role, facilitating the hydrogenation of *CO to *CHO and reducing NO_3_^−^ to *NH_2_ species.^[^ ^29^, ^31^ ^]^ The *NH_2_ species then rapidly couples with *CHO, followed by a slow hydrogenation process to form CH_3_NH* fragment. The produced CH_3_NH* further undergoes rapid coupling with *CHO to form a second C−N bond, then undergoes slow hydrogenation to produce (CH_3_)_2_N* fragment. This fragment subsequently rapidly couples with *CHO to produce DMF.

In the oxidative coupling process, dimethylamine and CH_3_OH readily undergo oxidative dehydrogenation to form (CH_3_)_2_N* and *CHO, respectively.^[^ ^28^ ^]^ Additionally, dimethylamine itself can be oxidized, undergoing C─N cleavage and dehydrogenation to generate *CHO. The resulting *CHO and (CH_3_)_2_N* then readily couple to generate DMF (Figure 6). The absence of hydrogenation steps and the rapid oxidative dehydrogenation and coupling process contribute to achieving high selectivity and activity for DMF production.

## 5 Conclusion

To summarize, our study presents an electrosynthesis of DMF using CO_2_ and NO_3_^−^ as building blocks, enabling a high production rate of 1.24 mmol h^−1^ g_cat_^−1^ for DMF using a hybrid Ag/Cu catalyst. We also achieved 28.6% FE_DMF_ from oxidative coupling using Ag/Cu catalyst. Then we use a retrosynthetic method to investigate the mechanism of DMF formation. Importantly, our investigation reveals that the hydrogenation of coupled intermediates rather than C−N coupling constitutes the limiting step in DMF synthesis. While previous reports have primarily focused on enhancing the efficiency of C−N coupling, our findings underscore the importance of directing attention toward promoting hydrogenation to facilitate the production of more complex C−N chemicals, beyond mere urea formation. This work expands the product and mechanism scope of C−N electrosynthesis from cheap CO_2_ and NO_3_^−^ building blocks, thereby enabling broadening the impact of this burgeoning field.

## Conflict of Interest

The authors declare no conflict of interest.

## Supporting information



Supporting Information

## Data Availability

The data that support the findings of this study are available from the corresponding author upon reasonable request.
