# Supplementary material for: Total Electrosynthesis of N, N‐Dimethylformamide From CO2 and NO3 −
Source: Adv Sci (Weinh). 2024 Nov 21;12(2):2414431. doi: 10.1002/advs.202414431 (PMC11727272; doi:10.1002/advs.202414431)
Supplement: Supplementary file 1 — Supporting Information [file ADVS-12-2414431-s001.pdf]

ADVANCED SCIENCE
Open Access

Supporting Information

for *Adv. Sci.*, DOI 10.1002/advs.202414431

Total Electrosynthesis of N, N-Dimethylformamide From CO_2 and NO_3^-

*Shuai Yan, Shuai Chen, Morgan McKee, Alexandre Terry, Ralf Weisbarth and Nikolay Kornienko**

Supporting Information

Total Electrosynthesis of N, N-Dimethylformamide from CO_2 and NO_3^-

*Shuai Yan, Shuai Chen, Morgan McKee, Alexandre Terry, Ralf Weisbarth, Nikolay Kornienko**

Methods

Chemicals and materials: KNO_3 (99%) was purchased from AppliChem GmbH. $KHCO_3$ (extra pure) and N, N-dimethylformamide (99.99%) were purchased from Thermo Fisher Scientific Inc. $NH_2OH{\cdot}HCl$ (>97%) and N-methylformamide (>99%) were purchased from TCI. $NH_3{\cdot}H_2O$ (28%) was purchased from VWR Chemicals. KNO_2 (97%), ^{15}N-KNO_3 (98%), methanol (99.8%), dimethylamine (40 wt% in H_2O), methylamine (40 wt% in H_2O), iodomethane (99%), and formamide (≥99%) were purchased from Sigma-Aldrich. Formic acid (88%) was purchased from Aldrich Chemical Company. The impedance of deionized (DI) water was 18.2 $M\Omega{\cdot}cm^{-1}$. All chemical reagents were used without further purification.

Preparation of electrodes: To prepare the gas diffusion electrode, the carbon cloth (MPL-WIS1011, Full Cell store) was first cleaned using deionized water to remove impurities, and dried using Ar gas. Next, we prepared 3 nm Cu catalyst by sputter coating (SPI Supplies, Model 14430) on the carbon cloth substrate at a deposition current of approximately 40 mA for 120 s, and prepared 4 nm Ag catalyst under similar conditions for 60 s. The hybrid 4 nm Ag/3 nm Cu catalyst was fabricated by sputtering Ag for 60 s followed by copper for 120 s at a deposition current of ~ 40 mA. Additionally, an 8 nm Ag/6 nm Cu catalyst was fabricated by sputtering silver for 120 s and copper for 240 s at the same deposition current. The Pt catalyst was prepared by sputter coating for 240 s.

Electrochemical measurements: The electrochemical C−N coupling measurements were conducted using an electrochemical station (SP150, EC-lab) in our homemade batch cells, which consisted of a gas diffusion electrode loaded catalyst as the cathode, a graphite rod as the anode, and an Ag/AgCl electrode as the reference. The flow rate of CO_2 gas was maintained at approximately 50 mL/min. All electrode potentials were converted to the reversible hydrogen electrode (RHE) reference scale using the following method:

$$E\ (vs.\ \text{RHE}) = E\ (vs.\ \text{Ag/AgCl}) + 0.059 \times \text{pH} + 0.197\ \text{V} \tag{1}$$

Product qualification: The gas-phase products were detected employing online gas chromatograph (Agilent Technologies, Inc., 8860 GC system). Liquid products were quantified by ^{1}H nuclear magnetic resonance (^{1}H-NMR) spectroscopy (Bruker 500 MHz) through a water suppression mode. The concentration of liquid products was quantified by the standard DMSO solution in D_2O. The following equation was used to determine the Faradaic efficiency (FE) and partial current density (j) of liquid product:

$$\text{FE} = \text{cVzF/Q} \times 100\%;\ \text{j} = \text{Q/t}_{\text{total}} \times \text{FE} \tag{2}$$

In this equation, z represents the number of electrons transferred, c and V correspond to the concentration of liquid products and the volume of the electrolyte, respectively. F denotes Faraday constant, and Q signifies the quantity of charges passed. t_{total} refers to the total time of the experiment.

To calculate the reaction rate of DMF formation, the following formulas were employed:

$$\text{Reaction rate (mmol h}^{-1}\ \text{cm}^{-2}) = \text{I} \times \text{t} \times \text{FE/(n} \times \text{F)} = \text{j} \times \text{t/(n} \times \text{F)} \tag{3}$$

$$\text{Reaction rate (mmol h}^{-1}\ \text{g}_{\text{cat}}^{-1}) = \text{I} \times \text{t} \times \text{FE/(n} \times \text{F} \times \text{m)} = \text{j} \times \text{t/(n} \times \text{F} \times \text{m)} \tag{4}$$

In this context, n represents the number of electrons transferred, t = 3600 s, and F denotes Faraday's constant (with units in C/mol). j and I represent the partial and total current densities (in mA cm^{-2}), while FE represents Faradaic efficiency. m denotes the mass loading (in g_{cat} cm^{-2}), as quantified by the ICP-OES results (**Table S1**).

Characterizations: Transmission electron microscopy (TEM) imaging was performed using a JEOL1400 TEM at an acceleration voltage of 120 kV. The catalyst sputtered on the carbon cloth was first scraped off with a blade and dispersed in ethanol. The TEM samples were then prepared by drop-casting the ethanol solution onto a carbon-coated copper grid. The inductively coupled plasma optical emission spectroscopy (ICP-OES) analysis was carried out using the PerkinElmer Optima 8300, equipped with a Meinhard nebulizer and a glass cyclonic spray chamber. Electrospray ionization (ESI) in positive mode was measured with an Orbitrap XL instrument from Thermo Fisher Scientific.

Electrochemical infrared spectroscopy study:

The *in-situ* attenuated total reflection surface-enhanced infrared absorption spectroscopy (ATR-SEIRAS) measurements were carried out using an electrochemical cell. A gold (Au) film approximately 60 nm thick was sputtered onto the reflection plane of a $ZnSe_2$ prism.

Subsequently, Ag and Cu were sputtered onto the aforementioned film, respectively. *In-situ* ATR–SEIRAS measurements were carried out using an optical system integrated into the spectroscopic chamber. Absorbance spectra were obtained by calculating $A = -\log(I_1/I_2)$, where I_1 and I_2 represent the irradiation intensity of the incident and reflective beams, respectively.

Electrochemical mass spectrometry (ECMS): *In-situ* ECMS testing was carried out in a homemade electrochemical cell using CO_2-saturated $KHCO_3$ and KNO_3 electrolytes. The working electrode consisted of a glassy carbon electrode sputtered with Ag and Cu, respectively. A graphite rod served as the anode, and an Ag/AgCl electrode was used as the reference electrode. Cyclic voltammetry was performed at a scan rate of 5 mV s^{-1} while simultaneously recording the mass spectrometry signals.

Statistical Analysis: Data in Figure 2e were obtained from over three independent samples and are presented as the mean ± standard deviation (SD). Statistical analyses were conducted using Origin 2018 software.

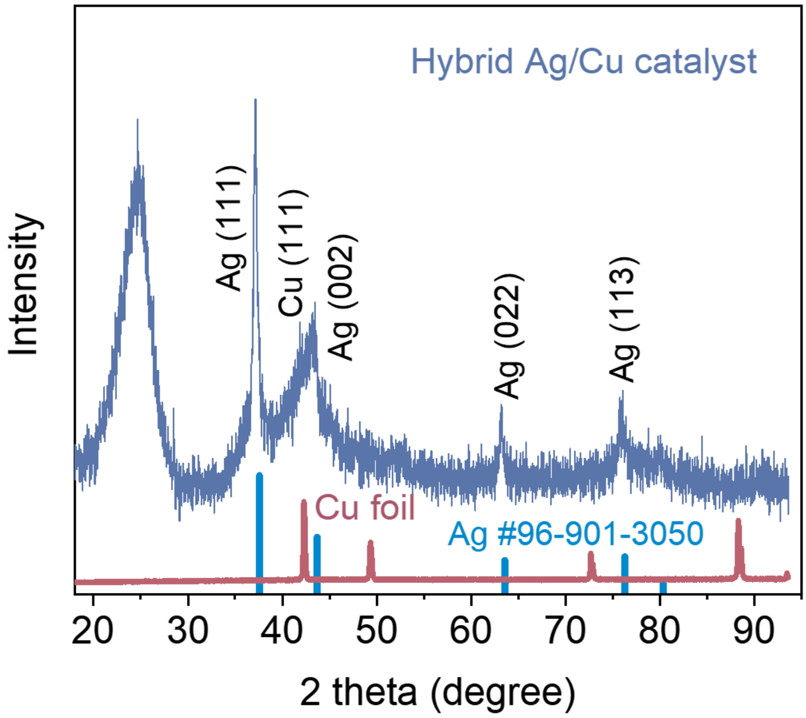

Figure S1. XRD patterns of hybrid Ag/Cu catalyst.

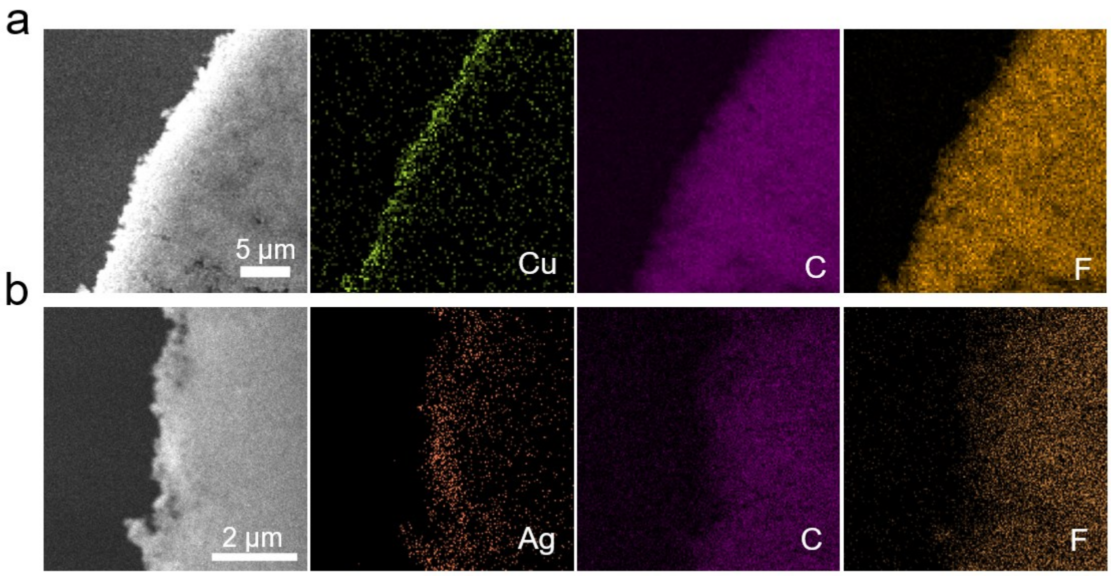

Figure S2. Elemental mapping of Cu (a) and Ag (b) after sputtering for 10 min and 4 min, respectively, across the cross-section of the carbon cloth.

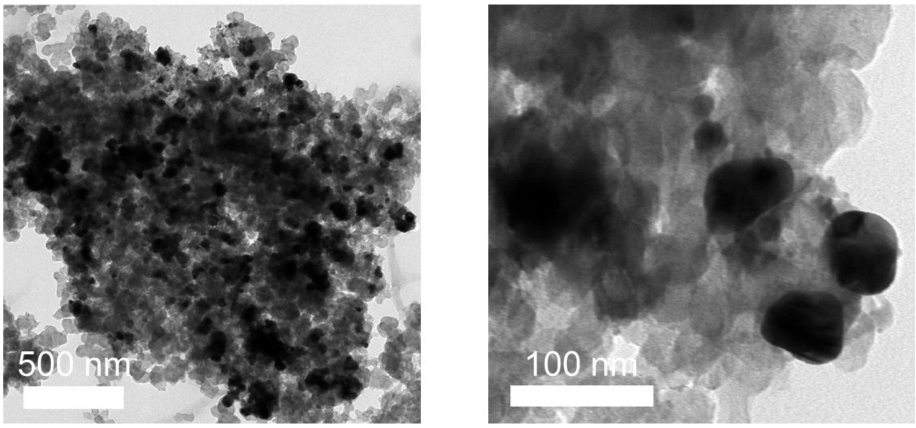

Figure S3. TEM images of hybrid Ag/Cu catalyst.

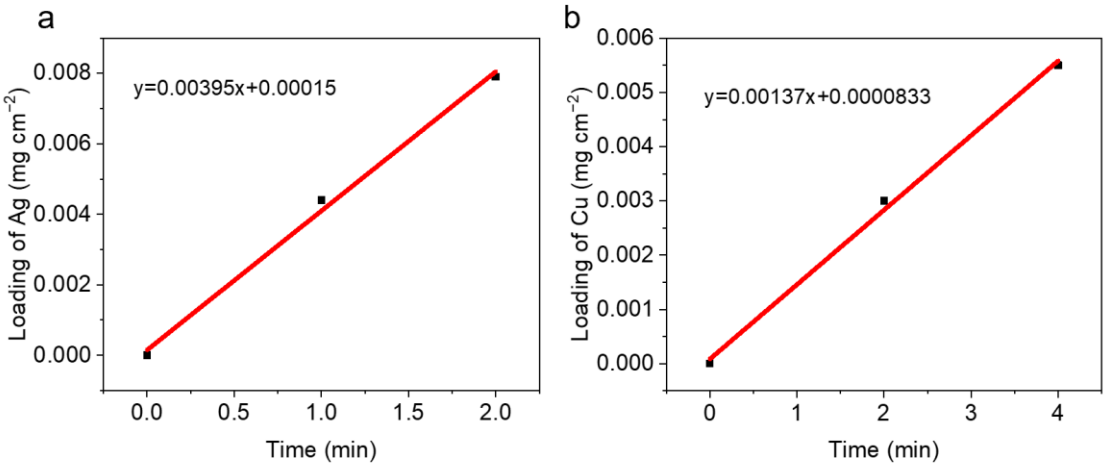

Figure S4. The thickness of Ag (a) and Cu (b) layers estimated from ICP-OES data as a function of sputtering time.

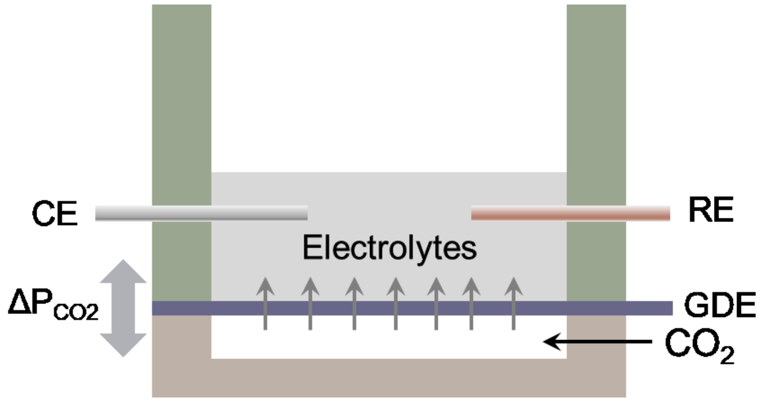

Figure S5. Scheme of our home-made gas diffusion half-cell with minimal electrolyte volume allows detection even at low yields, and a pressure gradient between the CO_2 flow side and the electrolyte side prevents flooding issues.

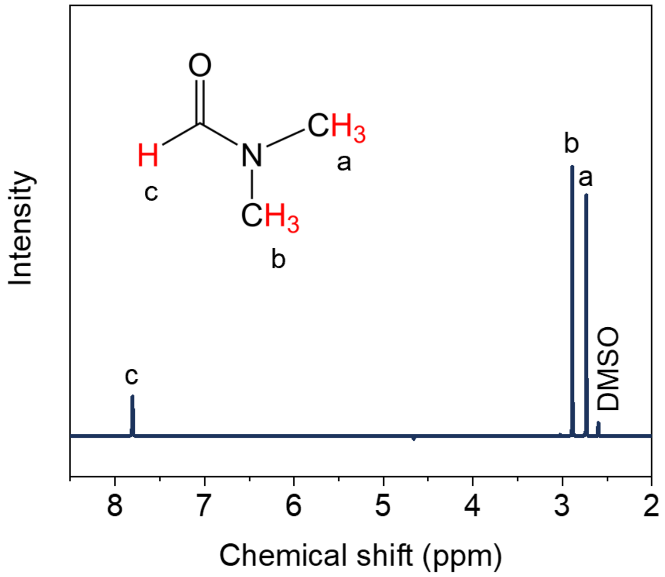

Figure S6. ^{1}H-NMR spectrum of standard DMF solution.

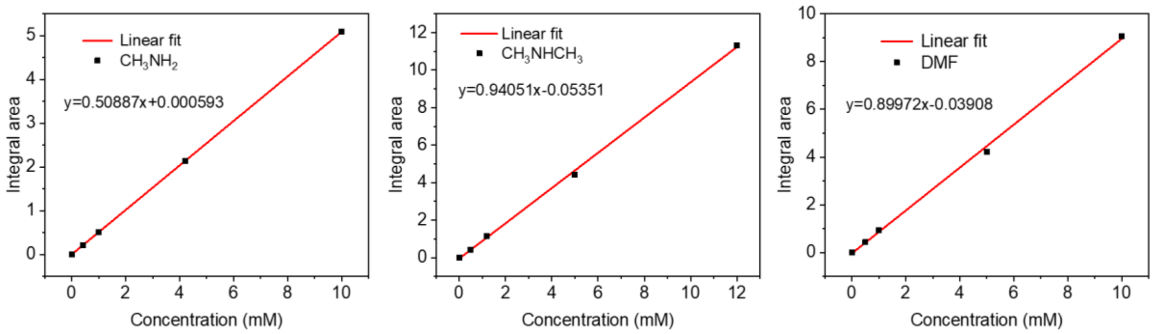

Figure S7. The calibration curve of methylamine, dimethylamine, and DMF products. The corresponding concentrations are linearly correlated with the integral area of their characteristic peaks.

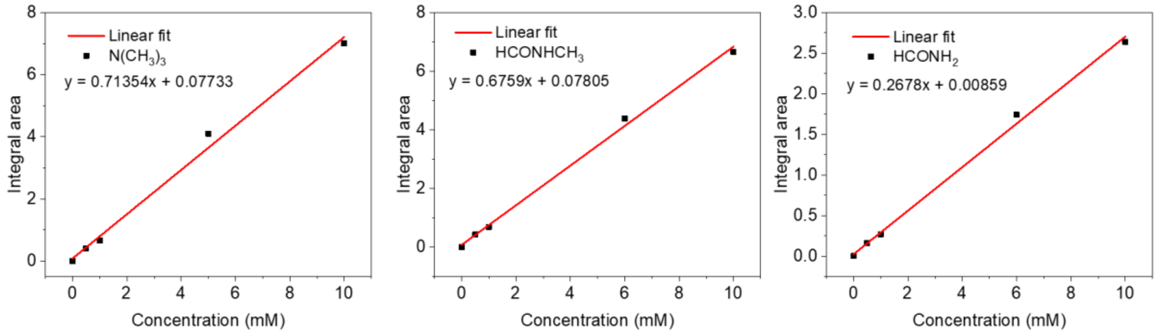

Figure S8. The calibration curve of trimethylamine, N-methylformamide and formamide products. The corresponding concentrations are linearly correlated with the integral area of their characteristic peaks.

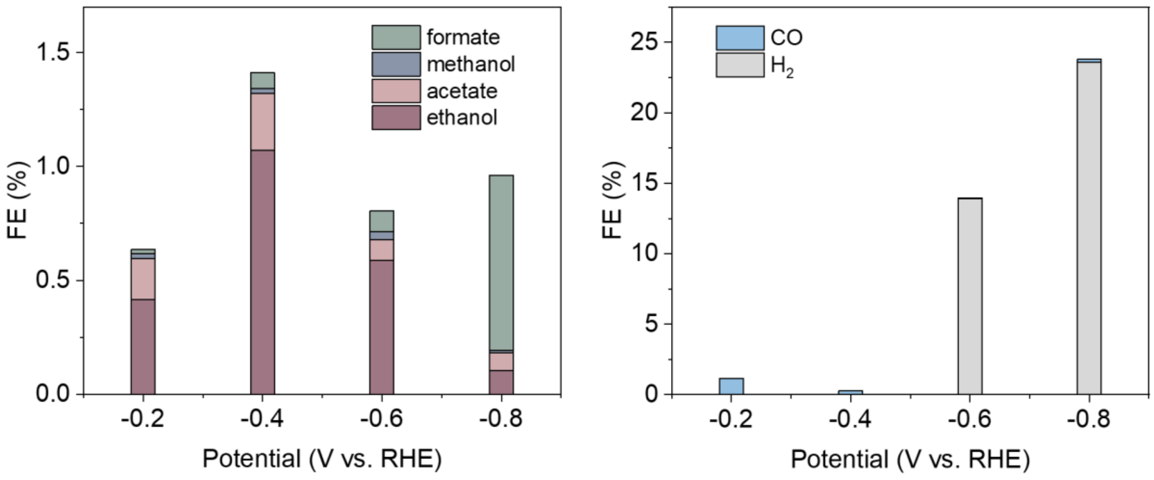

Figure S9. Potential-dependent FE values for liquid and gas products using an Ag/Cu hybrid catalyst. The overall FE of the reaction is low, likely due to the limited current at low overpotentials, resulting in low concentrations of products such as H_2, thus making accurate quantification difficult.

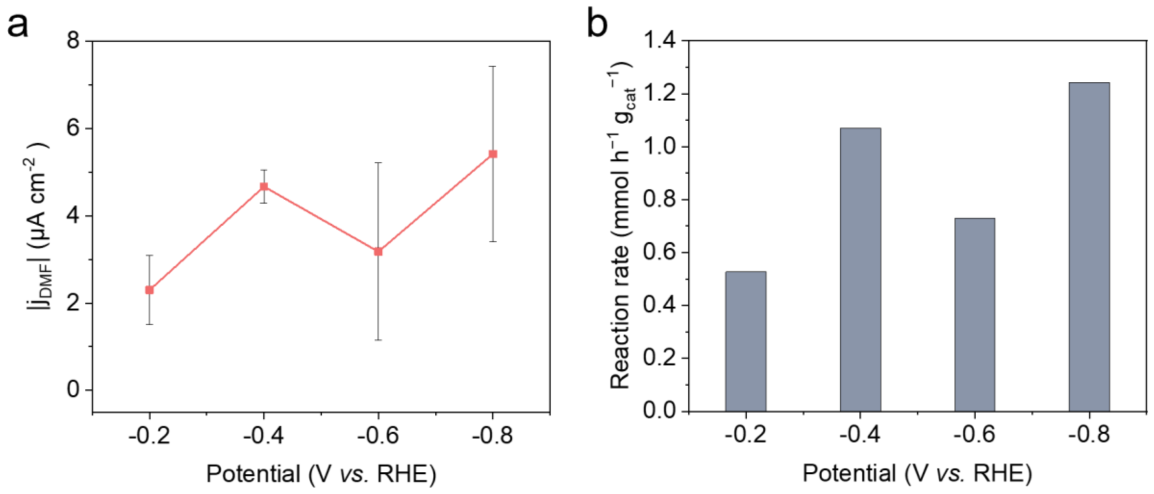

Figure S10. Partial current density (a) and reaction rate (b) for DMF using Ag/Cu catalyst at different potentials.

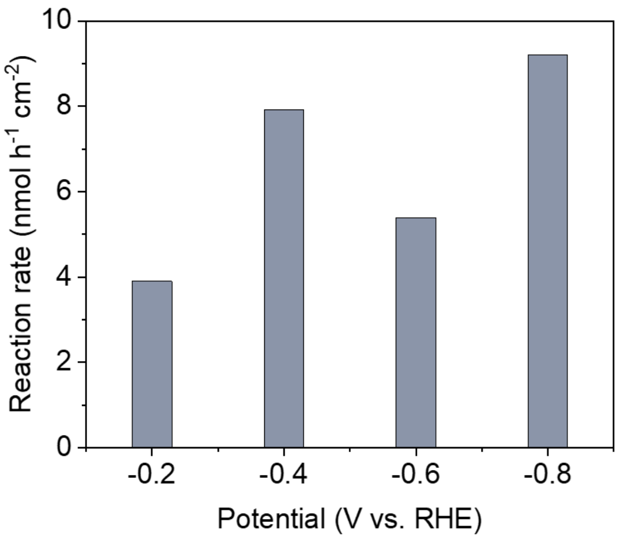

Figure S11. Reaction rate (nmol h^{-1} cm^{-2}) for DMF production using Ag/Cu catalyst at different potentials.

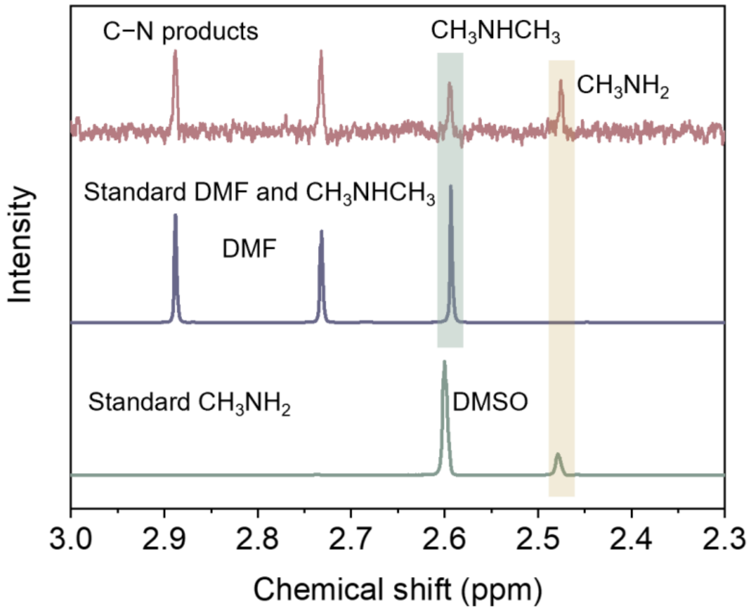

Figure S12. ^{1}H-NMR spectrum of methylamine, dimethylamine, and DMF products from C−N coupling. Bottom: ^{1}H-NMR spectrum of standard DMF, methylamine and dimethylamine solution.

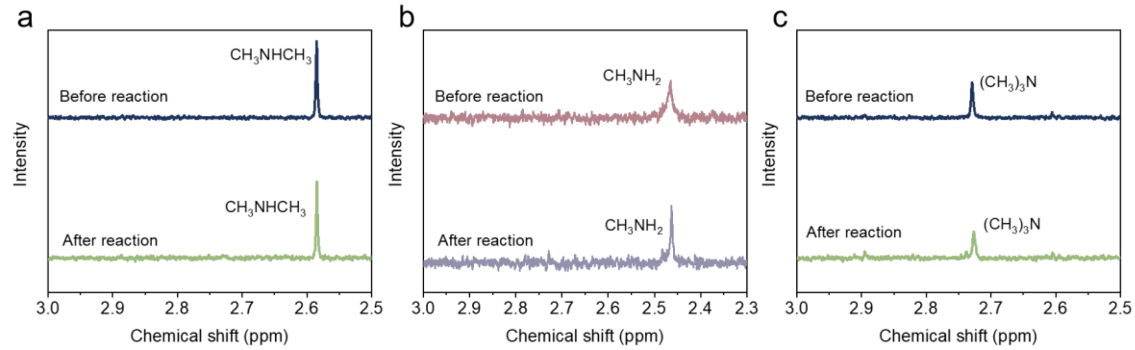

Figure S13. The ^{1}H-NMR results before and after the reaction at −0.4 V in the $KHCO_3$ and methylamine, dimethylamine and trimethylamine, respectively, using an undivided cell, where a graphite rod served as the anode and an Ag/Cu catalyst as the cathode.

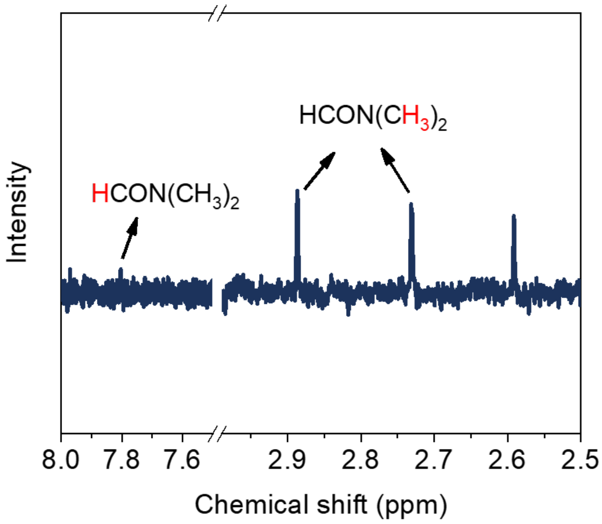

Figure S14. The ^{1}H-NMR results of C−N coupling at −0.4 V with the graphite rod and Ag/Cu catalyst separated by a cation exchange membrane.

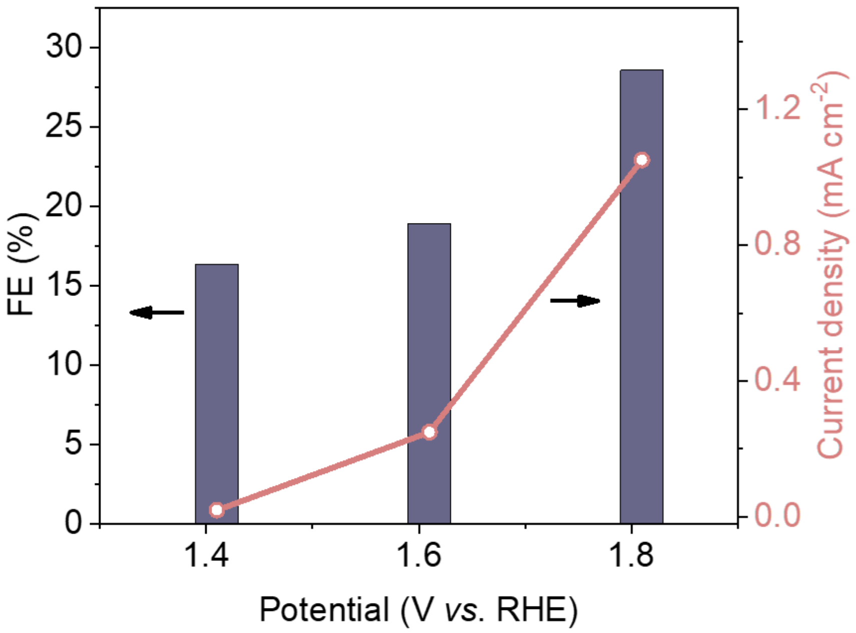

Figure S15 FE and j for DMF production from dimethylamine oxidation using Ag/Cu catalyst at different potentials.

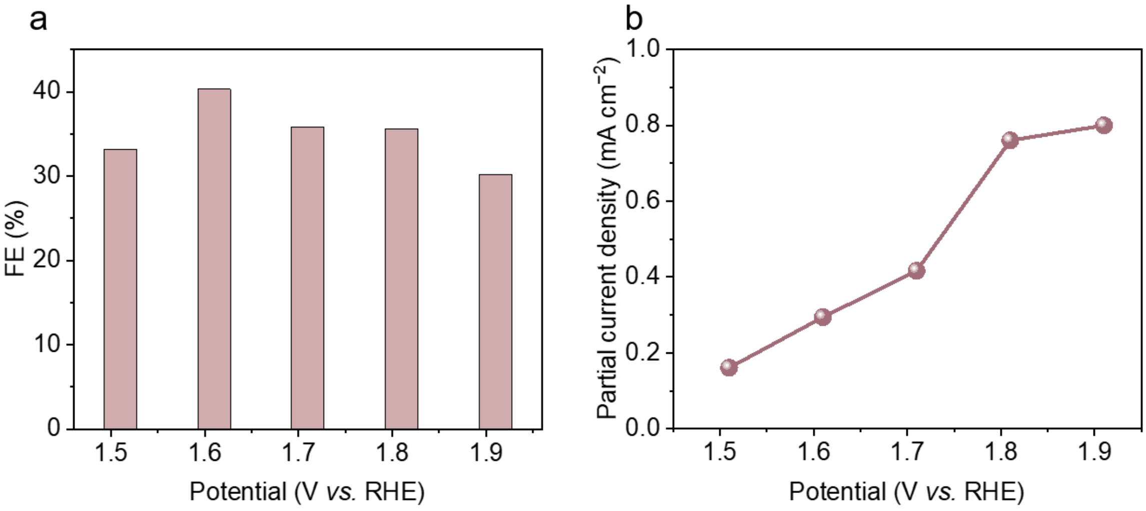

Figure S16. FE and j for DMF production from dimethylamine oxidation using Pt catalyst at different potentials.

.

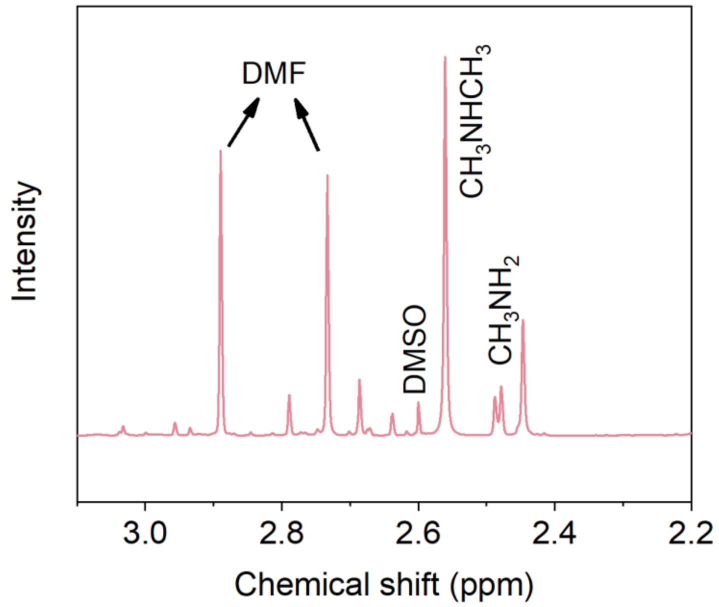

Figure S17. ^{1}H-NMR spectrum for C−N coupling using dimethylamine and $KHCO_3$ electrolytes at 1.61 V *vs.* RHE.

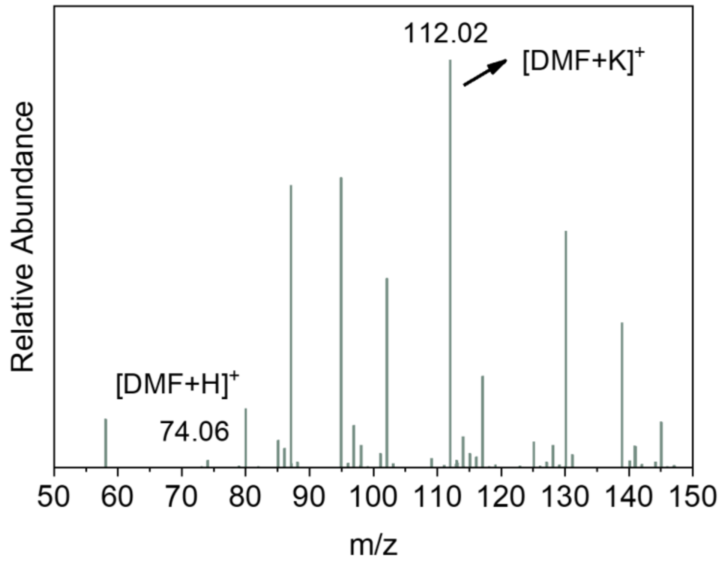

Figure S18. ESI(+) spectrum of DMF products from dimethylamine oxidation measured with an Orbitrap XL instrument. The *m*/*z* value of 74.06 corresponds to $[DMF + H]^+$; The *m*/*z* value of 112.02 corresponds to $[DMF + K]^+$.

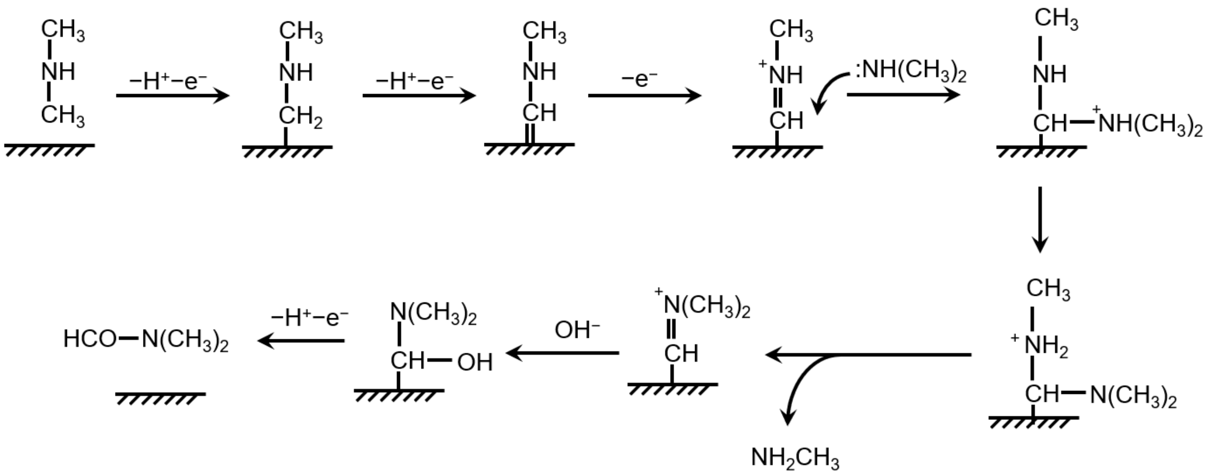

Figure S19. Possible reaction mechanism for the electrochemical oxidation of dimethylamine to produce DMF.

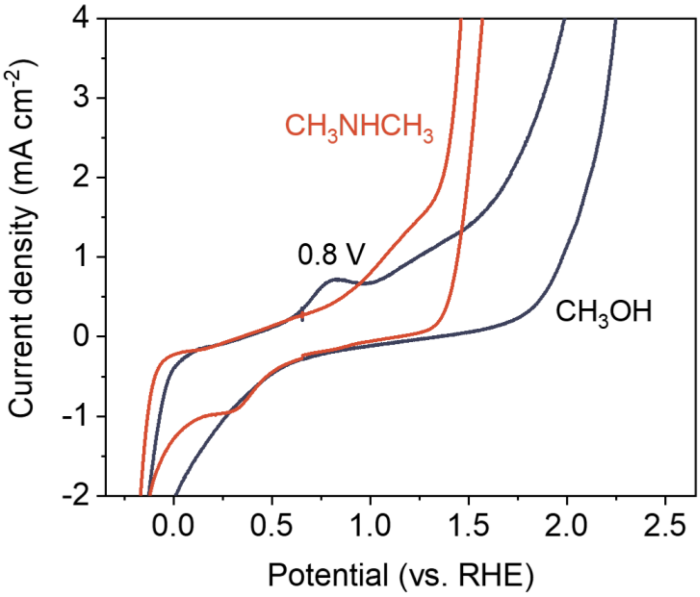

Figure S20. Cyclic voltammetry curves of methanol and dimethylamine oxidation using Pt catalyst, respectively, with the scan rate of 50 mV s^{-1}.

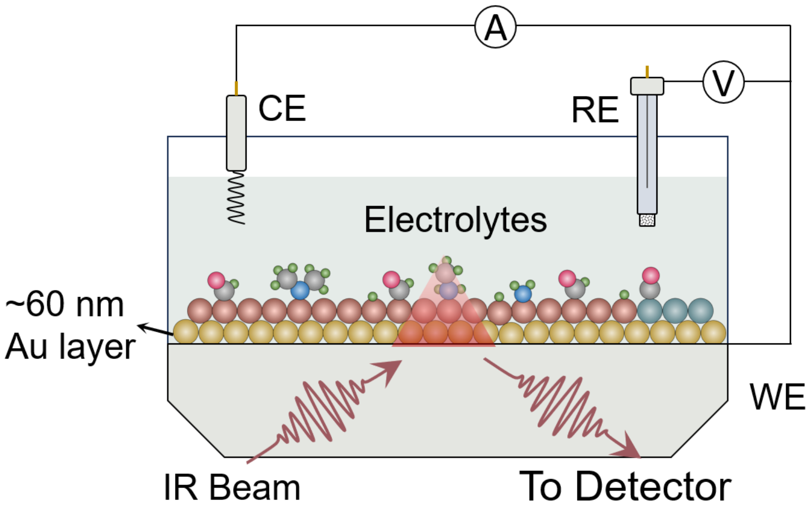

Figure S21. Schematic of *in-situ* ATR-SEIRAS measurement setup.

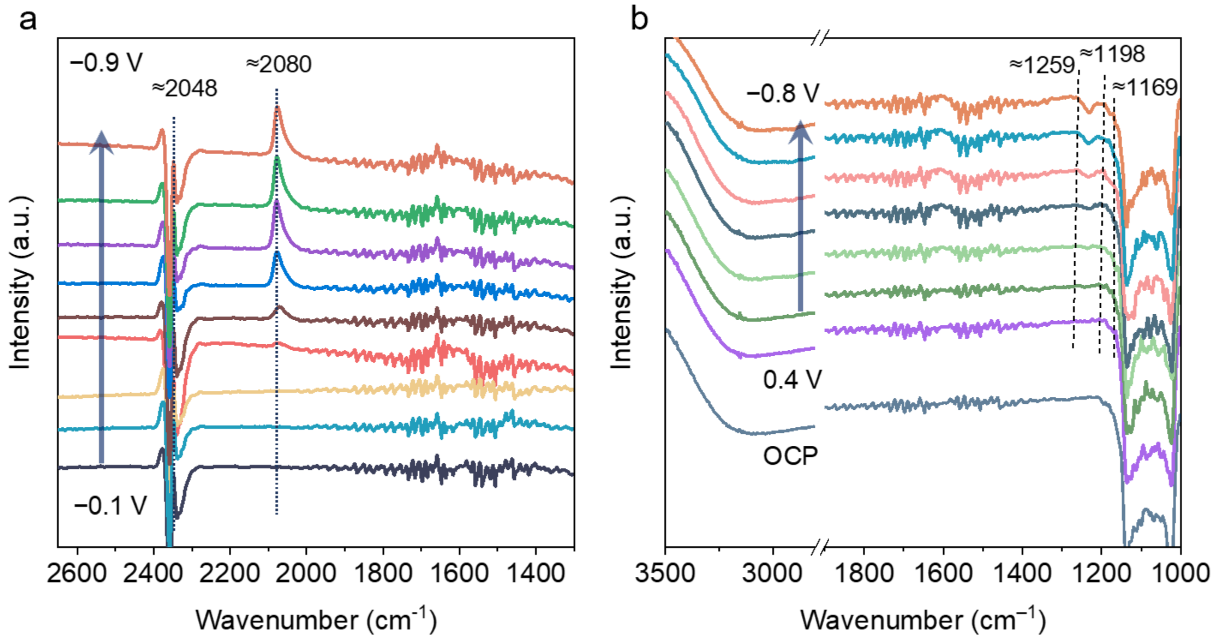

Figure S22. Potential-dependent *in-situ* ATR-SEIRAS on the Ag/Cu surfaces using CO_2-saturated $KHCO_3$ (a) and Ar-saturated KNO_3 solutions (b).

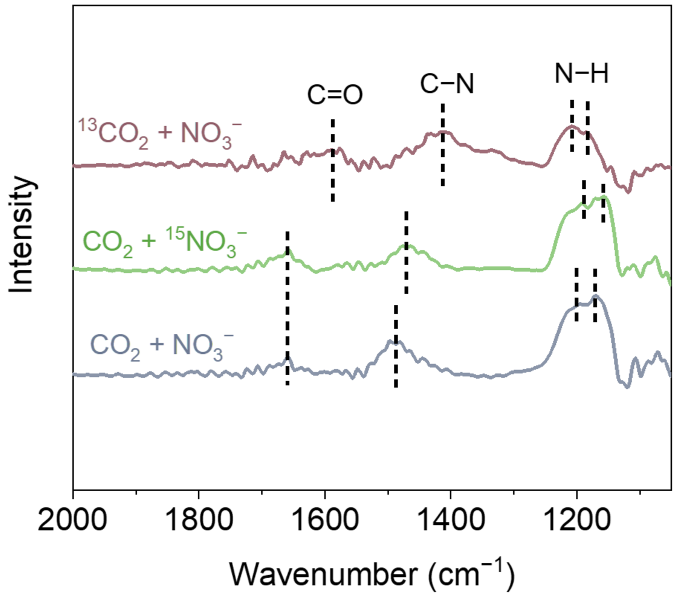

Figure S23. *In-situ* ATR-SEIRAS using Ag/Cu catalyst at −0.4 V in $^{13}CO_2$-$H^{13}CO_3^-$-NO_3^-, CO_2-HCO_3^--NO_3^- and CO_2-HCO_3^--$^{15}NO_3^-$ electrolytes.

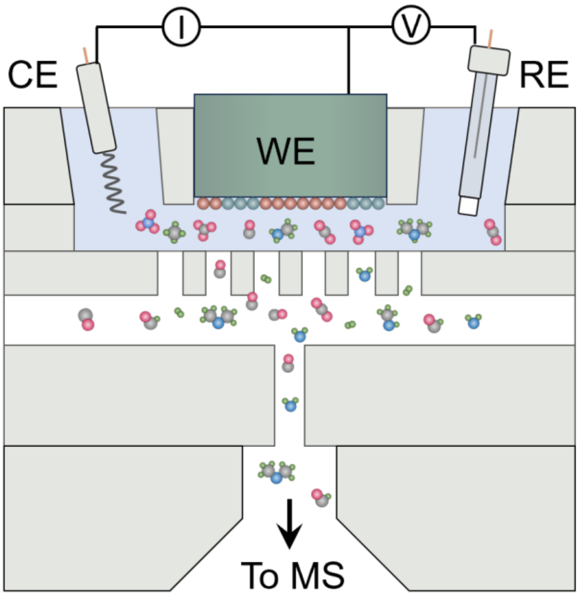

Figure S24. Schematic of *in-situ* ECMS measurement setup.

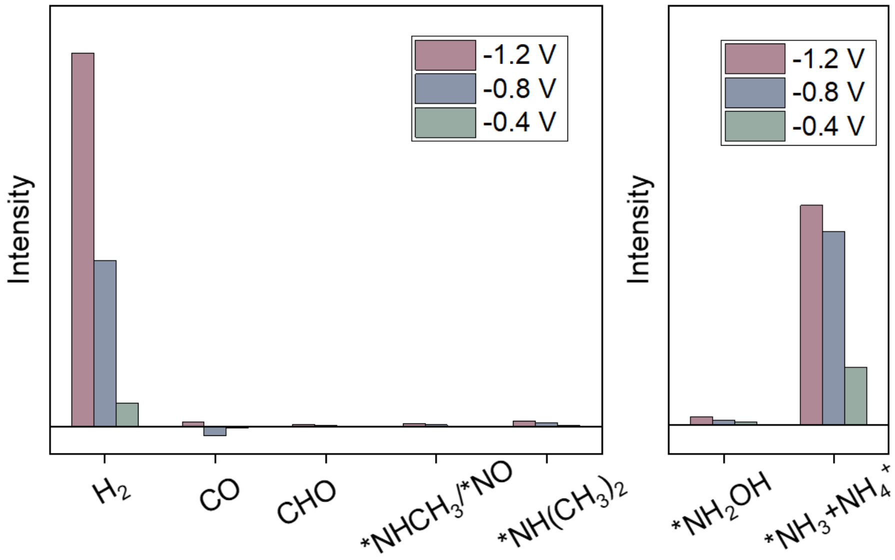

Figure S25 *In-situ* ECMS intensity at different potentials for the C−N coupling reaction over Ag/Cu catalyst.

Table S1. ICP-OES results of Ag/Cu catalysts.

Catalyst	Cu concentration (mg cm^{-2})	Ag concentration (mg cm^{-2})	Ag/Cu
4 nm Ag/3 nm Cu	0.0030	0.0044	1.47
8 nm Ag/6 nm Cu	0.0055	0.0079	1.44

Table S2. The list of control experiments conducted to elucidate the mechanistic pathway towards DMF.

Catalyst	Electrolytes	Gas	Potential (V *vs.* RHE)	FE (%)	Reaction rate (mmol g_{cat}^{-1} h^{-1})
None (GDE only)	0.02 M KNO_3 + 0.1 M $KHCO_3$	CO_2	−0.4	0	0
Ag/Cu	0.02 M KNO_3 + 0.1 M $KHCO_3$	Ar	−0.4	0	0
Ag/Cu	0.1 M $KHCO_3$	CO_2	−0.4	0	0
Ag/Cu	0.02 M KNO_3 + 0.1 M $KHCO_3$ + 0.02 M HCOOH	Ar	−0.4	0	0
Ag/Cu	0.02 M KNO_3 + 0.1 M $KHCO_3$ + 0.02 M HCHO	Ar	−0.4	0	0
Ag/Cu	0.02 M KNO_3 + 0.1 M $KHCO_3$ + 0.02 M CH_3OH	Ar	−0.4	0	0
Ag/Cu	0.02 M KNO_3 + 0.1 M $KHCO_3$	CO	−0.4	0.018	0.19
Ag/Cu	0.02 M KNO_2 + 0.1 M $KHCO_3$	CO_2	−0.4	0.053	0.30
Ag/Cu	0.02 M $NH_3 \cdot H_2O$ + 0.1 M $KHCO_3$	CO_2	−0.4	0.21	0.34
Ag/Cu	0.02 M NH_2OH + 0.1 M $KHCO_3$	CO_2	−0.4	0.11	0.37
Ag/Cu	0.02 M KNO_3 + 0.1 M $KHCO_3$	CO_2	−0.4	0.39	1.07
Ag/Cu	0.02 M CH_3I + 0.02 M KNO_3 + 0.1 M $KHCO_3$	CO_2	−0.4	0.6	1.12
Ag/Cu	0.02 M CH_3NH_2 + 0.1 M $KHCO_3$	CO_2	−0.4	0.74	1.20

Ag/Cu	Anode: 0.1 M $KHCO_3$<br>Cathode: 0.02 M CH_3NHCH_3 + 0.1 M $KHCO_3$	CO_2	−0.4	0.30	3.83
Ag/Cu	0.02 M $HCONH_2$ + 0.1 M $KHCO_3$	CO_2	−0.4	0.025	0.16
Ag/Cu	0.02 M $HCONHCH_3$ + 0.1 M $KHCO_3$	CO_2	−0.4	0.026	0.38

Due to the low solubility of CH_3I in H_2O, ethanol or acetone was added to increase its solubility.

Table S3. Retrosynthetic routes to distinguish the limiting step for DMF formation: hydrogenation *vs.* coupling.

Catalyst	Electrolytes	Gas	Potential (V *vs.* RHE)	Product	FE_{DMF} (%)	Reaction rate (mmol g_{cat}^{-1} h^{-1})
Ag/Cu	0.02 M KNO_3 + 0.1 M $KHCO_3$	CO_2	−0.4	CH_3NH_2	0.09	0.52
Ag/Cu	0.02 M $HCONH_2$ + 0.1 M $KHCO_3$	Ar	−0.4	CH_3NH_2	0.07	1.84
Ag/Cu	0.1 M CH_3I + 0.02 M KNO_3 + 0.1 M $KHCO_3$	CO_2	−0.4	CH_3NH_2	0.18	0.93
Ag/Cu	0.02 M $HCONHCH_3$ + 0.1 M $KHCO_3$	Ar	−0.4	CH_3NHCH_3	0.09	4.31
Ag/Cu	0.02 M $HCONH_2$ + 0.1 M $KHCO_3$	CO_2	−0.4	CH_3NHCH_3	0.14	1.21
Ag/Cu	0.02 M CH_3NH_2 + 0.1 M $KHCO_3$	CO_2	−0.4	CH_3NHCH_3	1.04	2.26
Ag/Cu	0.02 M CH_3NHCH_3 + 0.1 M $KHCO_3$	CO_2	−0.4	$(CH_3)_3N$	0.7	4.14
Ag/Cu	Anode: 0.1 M $KHCO_3$<br><br>Cathode: 0.02 M CH_3NHCH_3 + 0.1 M $KHCO_3$	CO_2	−0.4	DMF	0.30	3.83

Table S4. Oxidative C−N coupling routes to DMF using Ag/Cu catalyst.

Catalyst	Electrolytes	Potential (V *vs.* RHE)	FE_{DMF} (%)	Reaction rate (mmol g_{cat}^{-1} h^{-1})
Ag/Cu	0.1 M CH_3NHCH_3 + 0.1 M CH_3OH + 0.1 M $KHCO_3$	1.01	55	4.03
Ag/Cu	0.1 M CH_3NHCH_3 + 0.1 M $KHCO_3$	1.41	16.3	23.95
Ag/Cu	0.1 M CH_3NHCH_3 + 0.1 M $KHCO_3$	1.61	18.9	313.9
Ag/Cu	0.1 M CH_3NHCH_3 + 0.1 M $KHCO_3$	1.81	28.6	1324.8

Table S5. Oxidative C−N coupling routes to DMF using Pt catalyst.

Catalyst	Electrolytes	Potential (V *vs*. RHE)	FE_{DMF} (%)	Reaction rate (mmol g_{cat}^{-1} h^{-1})
Pt	0.1 M CH_3NHCH_3 + 0.1 M CH_3OH + 0.1 M $KHCO_3$	0.86	18.0	2.18
Pt	0.1 M CH_3NHCH_3 + 0.1 M $KHCO_3$	1.51	33.2	202.6
Pt	0.1 M CH_3NHCH_3 + 0.1 M $KHCO_3$	1.61	40.3	370.9
Pt	0.1 M CH_3NHCH_3 + 0.1 M $KHCO_3$	1.71	35.8	525.1
Pt	0.1 M CH_3NHCH_3 + 0.1 M $KHCO_3$	1.81	35.6	958.5
Pt	0.1 M CH_3NHCH_3 + 0.1 M $KHCO_3$	1.91	30.2	1008.4

Table S6. Possible band assignments of *in-situ* ATR-SEIRAS.

Wavenumber (cm^{-1})	Assignment	Intermediate	References
≈1169	N−H vibration	$^*NH_2$	
≈1198	N−H vibration	$^*NH_2OH$	*Chem Catal.*, **2023**, *3*, 100595;[S1] *Nat. Catal.*, **2023**, *6*, 402.[S2]
≈1259	N−O vibration	$^*NO_2$	
≈1497, ≈1436	C−N vibration	*C−N	*Nat. Chem.*, **2020**, *12,* 717;[S3] *ACS Energy Lett.*, **2023**, *8*, 3373.[S4]
≈1655	C=O stretching	*N−C=O	*Nat. Commun.*, **2022**, *13,* 5337;[S5] *Angew. Chem. Int. Ed.*, **2023**, *62*, e202210958.[S6]
≈2080	C≡O stretching	$^*CO_{atop}$	*Chem. Soc. Rev.*, **2010**, *39*, 4643;[S7] *Angew. Chem. Int. Ed.*, **2024**, *63*, e202400952.[S8]
≈2348	v_3 asymmetric stretching	CO_2	*J. Phys. Chem. A*, **2011**, *115*, 9854.[S9]
≈2927	C−H stretching	$^*CH_x$	*J. Phys. Chem. C*, **2018**, *122,* 21933;[S10] *J. Phys. Chem. C*, **2023**, *127,* 21695.[S11]

Table S7. Possible peak assignments of *in-situ* ECMS.

m/*z*	Intermediate	Reaction	References
2	H_2	HER	*Nat. Catal.*, **2023**, *6*, 807.[S12]
30	NO	NO_3RR	*Nat. Catal.*, **2023**, *6*, 402;[S2] *Adv. Funct. Mater.*, **2024**, 2401194.[S13]
33	NH_2OH	NO_3RR	
16	NH_2	NO_3RR	*J. Phys. D: Appl. Phys.*, **2002**, *35*, 665.[S14] *Int. J. Mass Spectrom. Ion Processes*, **1997**, *167*, 309.[S15]
17	NH_3	NO_3RR	*Nat. Catal.*, **2023**, *6*, 402.[S2]
18	NH_4^+	NO_3RR	*J. Chem. Phys., **2001**, 115*, 124.[S16]
28	CO	CO_2RR	*Nat. Catal.*, **2023**, *6*, 807.[S12]
29	CHO	CO_2RR	*Chem. Sci.*, **2011**, *2*, 1902.[S17] *Langmuir* **2008***, 24*, 4917.[S18]
30	CH_3NH	C−N coupling	*Electrochim. Acta*, **1992**, *37*, 759.[S19]
44	$(CH_3)_2N$	C−N coupling	*J. Electrochem. Soc.*, **1997**, *144*, 1127.[S20]

References

[S1] A. Guiet, A. Simonin, H. Bemana, H. Al-Mahayni, J. Li, K. Kuruvinashetti, R. Moury, A. Hémon-Ribaud, D. Chartrand, V. Maisonneuve, J. Lhoste, A. Seifitokaldani, D. Rochefort, N. Kornienko, *Chem. Catal.* **2023**, *3*, 100595.

[S2] S. Han, H. Li, T. Li, F. Chen, R. Yang, Y. Yu, B. Zhang, *Nat. Catal.* **2023**, *6*, 402.

[S3] C. Chen, X. Zhu, X. Wen, Y. Zhou, L. Zhou, H. Li, L. Tao, Q. Li, S. Du, T. Liu, D. Yan, C. Xie, Y. Zou, Y. Wang, R. Chen, J. Huo, Y. Li, J. Cheng, H. Su, X. Zhao, W. Cheng, Q. Liu, H. Lin, J. Luo, J. Chen, M. Dong, K. Cheng, C. Li, S. Wang, *Nat. Chem.* **2020**, 12, 717.

[S4] Y. Wang, S. Xia, J. Zhang, Z. Li, R. Cai, C. Yu, Y. Zhang, J. Wu, Y. Wu, *ACS Energy Lett.* **2023**, *8*, 3373.

[S5] X. Zhang, X. Zhu, S. Bo, C. Chen, M. Qiu, X. Wei, N. He, C. Xie, W. Chen, J. Zheng, P. Chen, S. P. Jiang, Y. Li, Q. Liu, S. Wang, *Nat. Commun.* **2022**, *13*, 5337.

[S6] J. Geng, S. Ji, M. Jin, C. Zhang, M. Xu, G. Wang, C. Liang, H. Zhang, *Angew. Chem. Int. Ed.* **2023**, *62*, e202210958.

[S7] B. L. Mojet, S. D. Ebbesen, L. Lefferts, *Chem. Soc. Rev.* **2010**, *39*, 4643.

[S8] Y. Wang, F. Yang, H. Xu, J. Jang, E. P. Delmo, X. Qiu, Z. Ying, P. Gao, S. Zhu, M. D. Gu, M. Shao, *Angew. Chem. Int. Ed.* **2024**, *63*, e202400952.

[S9] X. Zhang, S. P. Sander, *J. Phys. Chem. A* **2011**, *115*, 9854.

[S10] G.-K. Liu, S. Zou, D. Josell, L. J. Richter, T. P. Moffat, *J. Phys. Chem. C* **2018**, *122*, 21933.

[S11] Y. Wu, Z. Mao, T.-W. Jiang, X.-Y. Ma, L. Zheng, K. Jiang, W.-B. Cai, *J. Phys. Chem. C* **2023**, *127*, 21695.

[S12] H. Zhang, J. Gao, D. Raciti, A. S. Hall, *Nat. Catal.* **2023**, *6*, 807.

[S13] J. Su, K. Shi, B. Liu, Z. Xi, J. Yu, X. Xu, P. Jing, R. Gao, J. Zhang, *Adv. Funct. Mater.* **2024**, 2401194.

[S14] J. Jauberteau, I. Jauberteau, J. Aubreton, *J. Phys. D: Appl. Phys.* **2002**, *35*, 665.

[S15] M. J. Polce, Y. Kim, C. Wesdemiotis, *Int. J. Mass Spectrom. Ion Process.* **1997**, *167*, 309.

[S16] J. E. Flad, M. A. Everest, J. C. Poutsma, R. N. Zare, *J.Chem.Phys.* **2001**, *115*, 124.

[S17] K. Schouten, Y. Kwon, C. Van Der Ham, Z. Qin, M. Koper, *Chem. Sci.* **2011**, *2*, 1902.

[S18] K. Na, J. Jung, O. Kim, J. Lee, T. G. Lee, Y. H. Park, J. Hyun, *Langmuir* **2008**, *24*, 4917.

[S19] C.-P. Maschmeier, H. Baltruschat, *Electrochim. Acta* **1992**, *37*, 759.

[S20] A. Thon, D. Saulys, S. Safvi, D. Games, T. Kuech, *J Electrochem Soc.* **1997**, *144*, 1127.
